# The complete chloroplast genome sequence of *Clivia caulescens*

**DOI:** 10.1080/23802359.2021.1972050

**Published:** 2021-09-06

**Authors:** Hai-Hong Wu, Dan Li, Xiu-Li Feng, Ling Yue, Xing-Hua Zhao, Xu-Hui Chen

**Affiliations:** a Institute of Floriculture, Liaoning Academy of Agricultural Sciences, Shenyang, China; b College of Bioscience and Biotechnology, Shenyang Agricultural University, Shenyang, China

**Keywords:** *Clivia caulescens*, chloroplast genome sequence, phylogenetic tree

## Abstract

*Clivia caulescens* is an evergreen herbaceous flower with high ornamental value. In this study, we report its complete chloroplast genome sequence. The whole chloroplast genome was 158,149 bp in length, with a large single copy region (LSC, 86,250 bp), a small single copy region (SSC, 18,343 bp), and two inverted repeat regions (IRs, 26,778 bp). The overall GC content was 37.91%. There were 128 genes annotated, including 86 protein-coding genes, 34 tRNA genes, and eight rRNA genes. The phylogenetic tree showed that *C. caulescens* formed a monophyletic clade with *C. miniata*, *C. miniata* var. *aurea*, and *C. gardenii*.

*Clivia caulescens* R.A. Dyer 1943 belongs to the genus *Clivia* of Amaryllidaceae. As an evergreen herbaceous plant, *C. caulescens* is popular for its diverse colors, making it a prized ornamental. However, until now, the complete chloroplast genome sequence of *C. caulescens* has not been published. Herein, we report the complete chloroplast genome sequence of *C. caulescens* to provide a genomic resource and to clarify the phylogenetic relationship between *C. caulescens* and related taxa.

The sample of *C. caulescens* was collected from the Liaoning Academy of Agricultural Sciences (N41°48′33″, E123°34′53″), Shenyang, China. Total genomic DNA was isolated using a modified CTAB method (Doyle and Doyle 1987), and a specimen was deposited at the Institute of Floriculture of Liaoning Academy of Agricultural Sciences (contact person XH Zhao and email zhaohaihong1997@163.com) under the voucher number YJJZL04. The isolated genomic DNA was manufactured to create a shotgun library with an average of 400 bp, and sequenced on the Illumina NovaSeq platform with the 2 × 150 bp paired-end mode. The reads were then assembled by using GetOrganelle v1.6.2e (Jin et al. 2020) and annotated by the OGAP pipeline (https://github.com/zhangrengang/OGAP). The draft annotations were then adjusted manually.

The results showed that the complete chloroplast genome sequence of *C. caulescens* (GenBank accession No. MW660366) was a circular form with a typical four-segment of 158,149 bp in length, consisting of a large single-copy region (LSC, 86,250 bp), a small single-copy region (SSC, 18,343 bp), and two inverted repeat regions (IRs, 26,778 bp). The overall GC content of the genome was 37.91%. Additionally, the complete chloroplast genome of *C. caulescens* contained a total of 128 genes, including 86 protein-coding genes, 34 tRNA genes, and eight rRNA genes.

To better understand the phylogenetic relationship between *C. caulescens* and related species, the complete chloroplast genomes were downloaded from GenBank of 14 representative species of the family Amaryllidaceae, using *Lilium brownii* (Liliaceae) as an outgroup. Multiple alignment was carried out by MAFFT v7.471 software (Standley and Katoh 2013) and then trimmed with trimAl v1.2 (Capella-Gutiérrez et al. 2009). The phylogenetic tree was generated with IQ-TREE v1.6.5 (Nguyen et al. 2015), with the best-fit model of K3Pu + F+R3 and 1000 bootstrap replicates. The phylogenetic tree showed that *C. caulescens*, *C. miniata*, *C. miniata* var. *aurea*, and *C. gardenii* formed a monophyletic clade within Amaryllidaceae with 100% bootstrap values (Figure 1).

**Figure 1.**
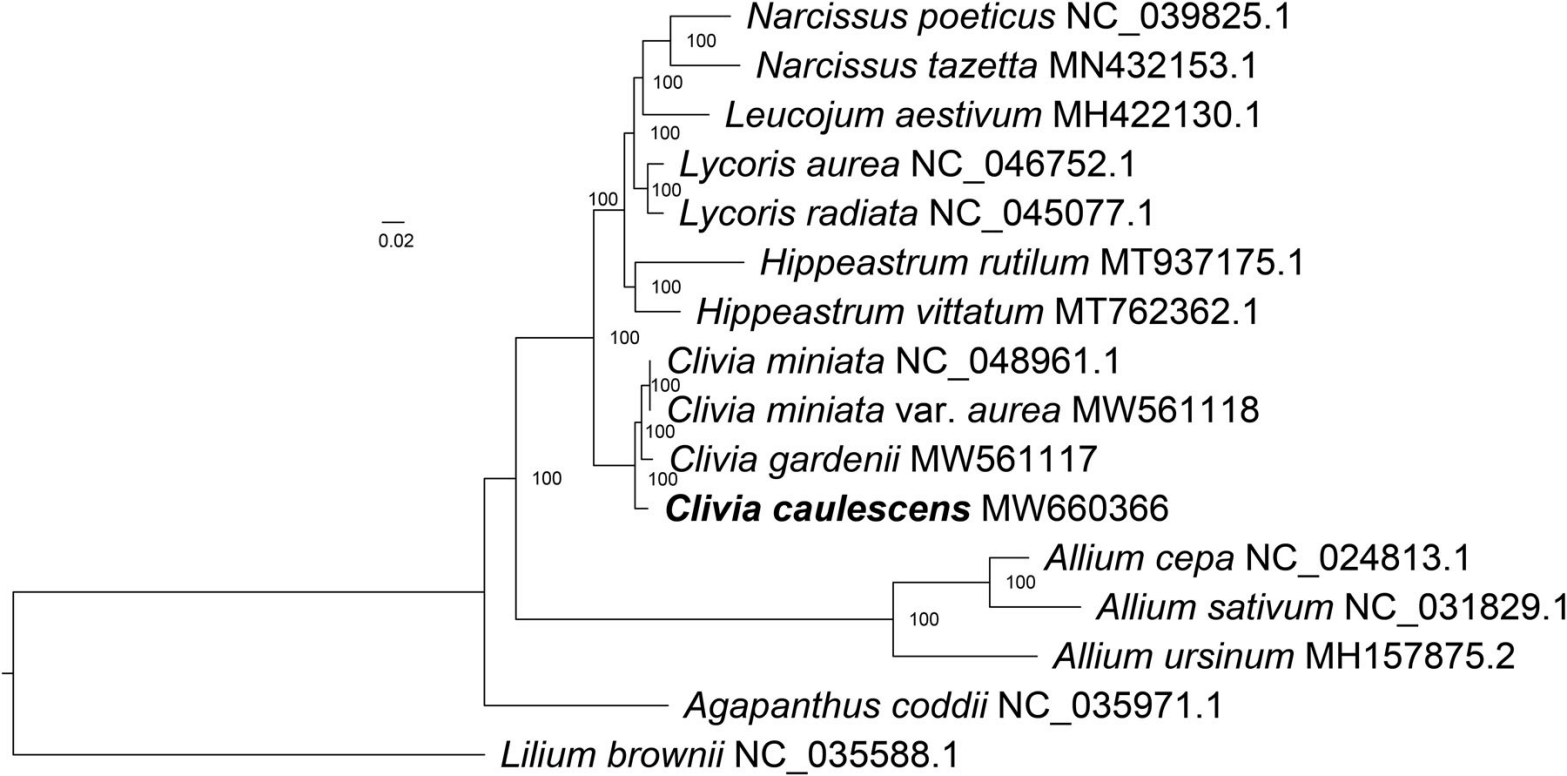
Maximum-likelihood (ML) tree based on the complete chloroplast genome sequences of *Clivia caulescens* and 14 other species, using *Lilium brownii* (Liliaceae) as the outgroup. Bootstrap values are indicated at the nodes.

## Data Availability

The data that support the analyses and results of this study are openly available in GenBank with accession number MW660366 (http://www.ncbi.nlm.nih.gov/). The associated BioProject, SRA, and Bio-Sample numbers are PRJNA704718, SRR13781248, and SAMN18053627, respectively.
